# Psychometric Properties of NASA-TLX and Index of Cognitive Activity as Measures of Cognitive Workload in Older Adults

**DOI:** 10.3390/brainsci10120994

**Published:** 2020-12-16

**Authors:** Hannes Devos, Kathleen Gustafson, Pedram Ahmadnezhad, Ke Liao, Jonathan D. Mahnken, William M. Brooks, Jeffrey M. Burns

**Affiliations:** 1 Laboratory for Advanced Rehabilitation Research in Simulation, Department of Physical Therapy and Rehabilitation Science, University of Kansas Medical Center, Kansas City, KS 66160, USA; pahmadnezhad@kumc.edu; 2 Department of Neurology, University of Kansas Medical Center, Kansas City, KS 66160, USA; kgustafson@kumc.edu (K.G.); wbrooks@kumc.edu (W.M.B.); jburns2@kumc.edu (J.M.B.); 3 Hoglund Brain Imaging Center, University of Kansas Medical Center, Kansas City, KS 66160, USA; kliao@kumc.edu; 4 University of Kansas Alzheimer’s Disease Center, University of Kansas Medical Center, Kansas City, KS 66160, USA; jmahnken@kumc.edu; 5 Department of Biostatistics & Data Science, University of Kansas Medical Center, Kansas City, KS 66160, USA

**Keywords:** event-related potentials, workload, reliability, working memory, mild cognitive impairment, dementia

## Abstract

Cognitive workload is increasingly recognized as an important determinant of performance in cognitive tests and daily life activities. Cognitive workload is a measure of physical and mental effort allocation to a task, which can be determined through self-report or physiological measures. However, the reliability and validity of these measures have not been established in older adults with a wide range of cognitive ability. The aim of this study was to establish the test–retest reliability of the National Aeronautics and Space Administration Task Load Index (NASA-TLX) and Index of Cognitive Activity (ICA), extracted from pupillary size. The convergent validity of these measures against event-related potentials (ERPs) was also investigated. A total of 38 individuals with scores on the Montreal Cognitive Assessment ranging between 17 and 30 completed a working memory test (*n*-back) with three levels of difficulty at baseline and at a two-week follow-up. The intraclass correlation coefficients (ICC) values of the NASA-TLX ranged between 0.71 and 0.81, demonstrating good to excellent reliability. The mean ICA scores showed fair to good reliability, with ICCs ranging between 0.56 and 0.73. The mean ICA and NASA-TLX scores showed significant and moderate correlations (Pearson’s r ranging between 0.30 and 0.33) with the third positive peak of the ERP at the midline channels. We conclude that ICA and NASA-TLX are reliable measures of cognitive workload in older adults. Further research is needed in dissecting the subjective and objective constructs of cognitive workload.

## 1. Introduction

Despite its incredible power and flexibility, there are limits to the brain’s capabilities. For example, working memory—the storage space that provides the foundation for higher-order cognitive functions—is inherently limited in its retention capability at any given time [1]. Performance on working memory tests is determined by the brain’s ability to allocate attention (mental effort) to the task and its available resources. Mental effort has traditionally been characterized as a direct measure of attention allocation to the task [2], although other studies have postulated that mental effort better reflects the readiness for resource expenditure [3]. Cognitive workload is the combined physical and mental effort put forth by an individual in response to the cognitive demand and time constraints of the task. If measured well, cognitive workload may have properties that offer relevant information beyond that provided by standard performance measures such as accuracy or response times of a task. When the cognitive workload required by the task is lower than the available cognitive resources, the task has the potential to be executed successfully. When the cognitive workload imposed by the task exceeds the available resources, task performance is expected to decrease [3]. Older age and age-related neurodegeneration may affect the availability of cognitive resources. With fewer resources available to attend to the task, older adults may show greater workload on a task compared to younger individuals [3]. This increased cognitive workload may reflect inefficient or compensatory use of neural resources to cope with the demand of the task. Some studies have suggested that this increased cognitive workload may serve as a predictor of cognitive decline [4].

Several techniques have been developed to measure cognitive workload, including questionnaires, performance outcomes, and physiological measures. The National Aeronautics and Space Administration Task Load Index (NASA-TLX) [5] is one of the most widely used questionnaires to determine cognitive workload [6]. This questionnaire relies on self-recall of cognitive workload and is typically administered after completion of the task. The NASA-TLX therefore does not provide continuous data but relies on the participant’s memory of events that have already occurred. Although the psychometric properties of the NASA-TLX have been established in a variety of disciplines such as aviation, military, driving, and skill acquisition [7,8], the reliability and validity of this instrument have not been tested in older adults with different levels of cognitive functioning.

Performance measures such as accuracy and response times are considered indirect measures of cognitive workload expenditure because they do not directly capture brain activity. Accuracy and response times on the *n*-back test are highly reliable performance measures of working memory in older adults [9]. The *n*-back is arguably the most ubiquitous working memory test across the age spectrum [10], yet previous studies have shown that the *n*-back test hosts an array of control processes, including speed of processing, storage, comparison processes, updating, keeping track, task mixing, task shifting, and resistance to interference [10,11,12].

Unlike performance measures, physiological measures can provide a continuous recording of brain activity in real time. Some studies have suggested that physiological changes may appear before the manifestation of symptoms in performance measures, thus providing a more sensitive measure of early cognitive decline [13,14,15]. In a systematic review, Ranchet et al. scrutinized the physiological changes resulting from increased cognitive workload in older adults with and without cognitive impairment. Increased hemodynamic and electrophysiological activity in the brain, smaller changes in systolic blood pressure, and increased pupillary dilation were observed in healthy older adults compared to younger adults, suggesting additional recruitment of neural resources to cope with task demands. In adults with neurodegenerative conditions, the inability to cope with task demand was even more apparent, resulting in not only an increase in hemodynamic, electrophysiological, and pupillary responses, but also worsening on performance measures [4].

Of those, the pupillary response is particularly interesting since it has been implicated in early tau accumulation in the locus coeruleus (LC) in Alzheimer’s disease (AD) [16]. Decreased neuronal density of the LC has been associated with cognitive decline in older adults, mild cognitive impairment, and AD [17]. The LC plays an essential role in the regulation of physiological arousal and cognition [18]. When activated, the LC sends inhibitory projections to the parasympathetic Edinger–Westphal nucleus, which, in turn, inhibits contraction of the pupillary sphincter muscle [19]. LC activity also triggers the sympathetic nervous system, resulting in activation of the pupillary dilator muscle [20]. A previous study found increased pupillary dilation in participants with single-domain mild cognitive impairment compared to cognitively normal participants, despite performance in the normal ranges [15]. Furthermore, participants with a genetic predisposition for AD showed greater relative pupillary size in tasks with high cognitive demand [21].

Two methods of examining pupillary response to cognitive workload have been reported. The task-evoked pupillary response (TEPR) compares the averaged raw pupillary diameter after stimulus onset to the averaged baseline pupillary diameter. Using raw pupillary dilation as a measure of cognitive workload poses some challenges, as the light reflex may confound extraction of the TEPR, especially in settings where the lighting of the surrounding environment or the luminosity of the screen cannot be entirely controlled [22]. Changes in camera angle and eye movements may also interfere with raw pupillary recording [20,23]. Nonetheless, previous studies found increased TEPR in individuals with elevated risk of AD [15,21]. An alternative to pupillometric baseline-related difference measures is the moment-to-moment pupillary diameter measurement. The Index of Cognitive Activity (ICA) and the Index of Pupillary Activity (IPA) are two moment-to-moment measures that calculate the rate of change of pupillary diameter, rather than the difference between averaged pupillary diameter after and before stimulus onset [24,25]. Both the ICA and IPA measures are based on the premise that pupils continuously undergo small fluctuations, even in steady illumination conditions [26]. An increase in abrupt discontinuities in the small oscillatory movements of the pupil reflects increased cognitive workload. These two measures of cognitive workload are claimed to successfully separate the pupillary response to cognitive workload from the light reflex. Furthermore, the ICA is claimed to be unaffected by changes in eye movements and sampling rate [27]. The ICA in particular has been used to investigate changes in cognitive workload in individuals at risk of cognitive impairment, including those with Parkinson’s disease, multiple sclerosis, and breast cancer [4,28,29,30,31,32]. Overall, the ICA seems to increase with cognitive demand, regardless of disease condition [29]. In addition, some studies report that individuals with increased risk of cognitive impairment show greater ICA compared to controls [30,32,33]. However, the reliability and validity of the ICA during working memory tasks in older individuals have not been established.

There is no gold standard for measuring cognitive workload. We selected the the third positive peak (P3) (or P300) event-related potential (ERP) as our criterion measure. The P3 is a positive peak at around 300 ms observed in visual or auditory working memory tasks. This component is considered a sensitive and reliable measure of cognitive workload, including in older adults with cognitive impairment [4,9,34,35,36]. EEG recordings show smaller ERP P3 amplitudes and longer P3 latencies in individuals with AD compared to controls [37,38]. The prolonged P3 latencies observed in patients with AD become particularly apparent in the cognitive domains of executive function, memory, and language [39]. With accuracy rates ranging between 70% and 94%, ERPs may also serve as useful predictors of conversion to AD [40]. The ability of the P3 ERP to discriminate between mild cognitive impairment (MCI) and AD [41] opens avenues for ERP as a potential screening tool for preclinical AD [42,43]. In addition, the P3 ERP is assumed to share the same neural origins in the LC as the pupillary response to cognitive workload, making this physiological response particularly suitable as a criterion measurement [44,45].

The aim of this study was to demonstrate the reliability of the NASA-TLX and the ICA and their convergent validity against the P3 ERP in older adults with a wide range of cognitive ability.

## 2. Materials and Methods

### 2.1. Participants

In this test–retest reliability study, 38 right-handed participants were recruited from the University of Kansas Alzheimer’s Disease Center between 3 May 2018 and 10 March 2020. Participants were included in the study if they (1) signed informed consent; (2) were 65 years of age or older; and (3) were able to understand the instructions in English. Exclusion criteria were (1) current use of steroids, benzodiazepines, or neuroleptics; (2) history of any substance abuse, (3) history of a psychiatric or neurological disorder other than MCI or AD; and (4) vision problems that cannot be resolved by corrective lenses.

Each participant had previously undergone an amyloid PET scan of the brain. Intravenous florbetapir F-18A was administered in a GE Discovery ST-16 PET/CT scanner to assess the cerebral amyloid burden. The Standard Uptake Value Ratio for six regions of interest was calculated using MIMneuro software (MiM Software Inc., Cleveland, OH, USA) by normalizing the Aβ PET image to the entire cerebellum. Each participant was categorized into one of three groups: (1) cognitively normal (CN), non-elevated or Aβ−; (2) CN, elevated or Aβ+; or (3) MCI/AD. The Clinical Dementia Rating Scale (CDR) staging was first reviewed to determine CN (CDR = 0) and cognitively impaired (CDR > 0) individuals. Next, performance on cognitive testing and additional clinical information (i.e., MRI) were considered to arrive at consensus on the classification (CN, MCI, AD) and etiologic diagnosis. The recommendations from the National Institute on Aging and the Alzheimer’s Association workgroup were used to categorize participants into Aβ− and Aβ+ [46]. The protocol for the determination of amyloid elevation is described elsewhere [47]. The average (standard deviation) time between administration of the PET scan and pupillometry/EEG assessment was 1090 (479) days. Sixteen participants were cognitively normal older adults with no elevated amyloid PET scans (Aβ−), 16 were cognitively normal with elevated amyloid PET scans (Aβ+), and 6 had a clinical diagnosis of MCI or AD. Participants completed their two-week follow-up session 16 ± 7 days after the first session. Each session lasted about 60 min, including rest breaks.

This study was approved by the Institutional Review Board of the University of Kansas Medical Center (#4461). All participants read and signed the informed consent form.

### 2.2. Procedure

#### 2.2.1. Demographic and Clinical Information

Age, sex, and education were recorded. General cognitive functions were evaluated using the Montreal Cognitive Assessment (MOCA) [48]. Scores on the MOCA range between 0 and 30.

#### 2.2.2. *n*-Back Test

In this study, the 0-back, 1-back, and 2-back tests were administered. The 0-back test is essentially a memory search task of sustained attention and often used as a control condition [10,12]. Participants were instructed to press the button as soon as the letter “X” amongst a series of distracter letters appeared on the screen. The 1-back test requires the participant to passively store and update information in working memory. In this test, participants had to press the button if the current letter was the same as the previous letter. The 2-back test requires continuous mental effort to update information of new stimuli and maintain representations of recently presented stimuli in short-term memory [49]. Participants were instructed to press the button when the current letter was the same as the letter presented two places before.

An extensive description of the 7-min test is provided elsewhere [9]. In short, each *n*-back test comprised 180 trials, including 60 (33.3%) target trials and 120 (66.7%) nontarget trials. The display time of each letter was 500 ms, followed by a blank interstimulus interval of 1700 ms with a random jitter of 50 ms. The maximum response time was 2150 ms. The participants practiced before the task.

#### 2.2.3. National Aeronautics and Space Administration Task Load Index

The NASA-TLX is one of the most frequently used self-reported questionnaires on cognitive workload. Six items of mental demand, physical demand, temporal demand, effort, performance, and frustration provide a comprehensive measure of cognitive workload [5]. Each item is scored on a visual analogue scale ranging from 0 to 100 in 5-point increments. NASA-TLX was administered immediately after each *n*-back test. The mean score of the six subscales was computed for each of the conditions and for each subject. In contrast to the original calculation [5], we did not attribute weights to each of the components since the unweighted average produced better sensitivity and reliability than the weighted average [50].

#### 2.2.4. Index of Cognitive Activity

While doing the *n*-back test, participants wore mobile eye tracking glasses (SMI ETG 2, Sensomotoric Instruments, Teltow, Germany). Pupillary size was recorded in real time at 60 Hz using infrared cameras for both the left and right eyes. Pupillary data were analyzed using Eyeworks (Eye Tracking, Inc., Solana Beach, CA, USA). The software analyzed the change in pupillary size for each eye throughout each *n*-back test. Potential artifacts from lighting and eye movements were minimized by using constant room lighting and having the participants focus on the screen. However, even under constant lighting conditions, the pupil continues to oscillate irregularly. Therefore, we transformed the raw pupil data to Index of Cognitive Activity (ICA) scores [24,51]. The ICA discriminates rapid, small bursts in pupillary dilation due to cognitive workload from slower, larger-amplitude changes in pupillary size due to the light reflex by decomposing the raw pupillary size into different wavelets of high- and low-frequency components of the signal [24]. Although the exact computation of the ICA is patented, the IPA shows a similar approach of computation of the pupillary response to cognitive workload [25]. The ICA has a low autocorrelation at a lag of 100 ms and almost no autocorrelation at a lag of 200 ms [52]. The ICA is calculated by dividing the number of rapid small pupillary dilations per second by the number of expected rapid pupillary dilations per second. The values are then transformed using the hyperbolic tangent function. Blinks are factored out by linear interpolation of adjacent time spans to produce continuous values ranging between 0 and 1 [24].

The average percentage of missing data collected from the eye tracker ranged between 0.87% and 2.24%. Three participants had more than 50% missing ICA values in one or more tests. These values were excluded from the analyses. The mean ICA values of the left and right eyes were included as outcome measures.

#### 2.2.5. P3 Event-Related Potential

Continuous electro-encephalograms (EEGs) were recorded at 1000 Hz using an Electrical Geodesics high-density system (Magstim EGI, Eugene, OR, USA) with 256 scalp electrodes. The start and end of the task were time-stamped and synchronized with the EEG and ICA recordings. The EEG recordings were filtered from 0.50 to 30 Hz using EGI software. All other EEG processing was done in EEGLab [53] and in ERPLab [54]. EEG data were online referenced to Cz and offline re-referenced to the average of mastoids. Cz was interpolated using the surrounding five channels. Independent component analysis was employed to separate brain activity from ocular, muscular, or cardiovascular artifacts. Signals from bad electrodes were removed and interpolated with the data of the surrounding electrodes. Continuous EEG data were segmented into epochs ranging between −100 and 1000 ms of stimulus onset. Each epoch was baseline corrected using the prestimulus interval. Scalp locations and measurement windows for the P3 ERP were based on their spatial extent and latency after inspection of the grand average waveform of the task effect. The task effect was calculated by subtracting the average ERP elicited from the targets from the average ERP elicited by non-targets for each participant. The P3 component time window was established between 200 ms and 400 ms for all three tests. Because of the prefrontal cortex’s involvement in working memory, we identified a priori Fz as the main channel, but we also report the results of the midline electrodes Cz and Pz. No participants were removed from the analyses because of artifacts. The P3 peak amplitude of the task effect was considered the main outcome measure to test convergent validity against, but we also calculated the P3 peak latency. The P3 peak amplitude and, to a lesser extent, the P3 peak latency are reliable measures of cognitive workload [9].

### 2.3. Data Analysis

Descriptive analysis, including the mean (standard deviation, SD) and frequency count of participants’ general, performance, NASA-TLX, ICA, and ERP data, was performed as appropriate. Intra-class correlation coefficients (ICCs) were used to calculate the test–retest reliability of ICA values and NASA-TLX scores. ICCs were computed as the between-subject variance divided by the total (between + within) variance [55]. ICC values less than 0.40 were considered poor; values between 0.40 and 0.59 were considered fair, values between 0.60 and 0.74 were considered good, and values between 0.75 and 1.00 were considered excellent [56]. Bland–Altman plots were used to visualize the measurement precision of NASA-TLX scores and ICA values across the test moments [57]. Intersubject stability according to subject rankings was calculated using the Pearson r correlation coefficient. The minimal detectable change at a 90% confidence interval (MDC_90_) provides a clinically useful indication of absolute reliability and reflects whether an observed change score is above that expected due to measurement error [58]. MDC_90_ was calculated as 1.645 × standard error of measurement (SEM) × $\sqrt{2}$ where SEM = SD_(first test)_ × $\sqrt{\left(1-\mathrm{ICC}\right)}.$ The Kolmogorov–Smirnov test was employed to test the normality of our data distribution in addition to visualization of Q–Q plots. All analyses were done using SAS Enterprise 8.2 and SAS 9.4 software. The threshold of significance was set at α = 0.05.

## 3. Results

### 3.1. Participant Characteristics

The participants (*n* = 38; 23 (61%) women) were, on average, 73.81 (5.23) years old and scored 26.97 (2.91) on the MOCA scale. The MOCA scores of participants ranged between 17 and 30.

### 3.2. Test–Retest Reliability of NASA-TLX

Overall, the NASA-TLX scores showed great consistency across the two test moments. The ICC scores ranged between 0.71 for 2-back and 0.81 for 0-back, demonstrating good to excellent reliability (Table 1). Pearson r correlations ranged between 0.55 for 2-back and 0.68 for 0-back, indicating strong intersubject stability. The MDC of the NASA-TLX ranged from 15.82 points on the 0-back test to 24.33 points on the 2-back.

Group analysis according to diagnosis (Aβ−; Aβ+; MCI/AD) did not affect the magnitude of ICC (Supplementary Table S1). The ICC values ranged from 0.50 (1-back) to 0.70 (0-back) for the Aβ− group; 0.73 (2-back) to 0.95 (0-back) for the Aβ+ group; and 0.87 (0- and 1-back) to 0.88 (2-back) for the MCI/AD group.

**Figure 1:**
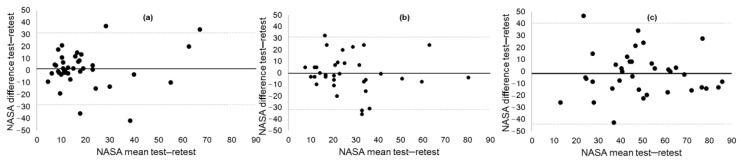
Bland–Altman plots of (**a**) 0-back NASA-TLX; (**b**) 1-back NASA-TLX; and (**c**) 2-back NASA-TLX.

The Bland–Altman plots showed equal spread of data around the mean (Figure 1). However, the spread of the NASA-TLX difference scores (limits of agreement, LOAs) was slightly larger in the 2-back test (95% confidence interval (CI), −43.88 to 40.63) compared to the 0-back (95% CI, −28.37 to 31.28) and 1-back (95% CI, −31.78 to 30.95) tests.

### 3.3. Test–Retest Reliability of ICA

All ICC values of the ICA measure were statistically significant (Table 1). The ICC values were highest for the left mean ICA (ICC = 0.73) and lowest for the right mean ICA (ICC = 0.56) in the 1-back test. All ICC values produced fair to good reliability. The Pearson *r* correlations were highest for the left mean ICA (*r* = 0.58) and lowest for the right mean ICA (*r* = 0.39) in the 1-back test. The MDC of ICA values ranged from 0.20 (in 0-back and 1-back) to 0.25 (in 2-back) for the right eye.

**Figure 2:**
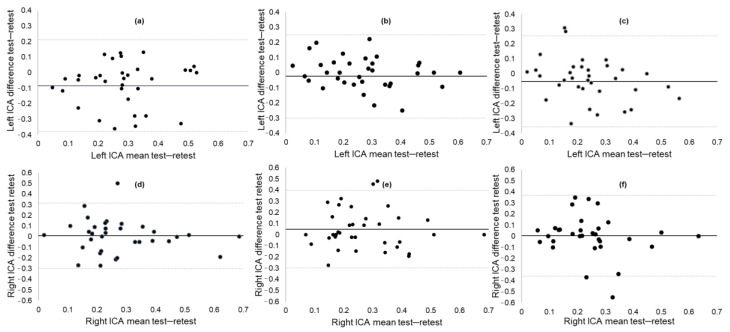
Bland–Altman plots of (**a**) the left eye 0-back ICA mean; (**b**) the left eye 1-back ICA mean; (**c**) the left eye 2-back ICA mean; (**d**) the right eye 0-back ICA mean; (**e**) the right eye 1-back ICA mean; and (**f**) the right eye 2-back ICA mean. ICA, Index of Cognitive Activity.

Figure 2 shows the Bland–Altman plots for each test. Plot (a) displays a negative mean ICA difference of the left eye of −0.08, indicating a slight decrease in mean ICA at the two-week follow-up compared to the baseline assessment. Plots (b) to (f) demonstrate equal distribution of the data around zero, indicating no bias in the results and no heteroscedasticity within the data.

The ICC values were similar across groups, except for the 0-back and 2-back left ICA means, which were substantially lower in the MCI/AD groups compared to the Aβ− and Aβ+ groups (Supplementary Table S1). The ICC values ranged between 0.60 (2-back right ICA mean) and 0.80 (0-back right mean ICA) for the Aβ− group; 0.42 (1-back right ICA mean) and 0.88 (1-back left ICA mean) for the Aβ+ group; and −2.44 (2-back left ICA mean) and 0.91 (1-back right ICA mean) for the MCI/AD group.

### 3.4. Convergent Validity of NASA-TLX

0-back: There was a trend such that higher total scores on the NASA-TLX correlated with increased peak P3 latency at channel Fz (*r* = 0.31; *p* = 0.06). On the item level, higher performance scores on the NASA-TLX correlated with lower peak P3 amplitude at channel Fz (*r* = −0.35, *p* = 0.04). The temporal demand item of the NASA-TLX correlated with peak P3 amplitude at channel Cz (*r* = 0.37; *p* = 0.03).

1-back: Higher NASA-TLX total scores correlated with increased peak P3 latency at Pz (*r* = 0.32; *p* = 0.05). On the item level, mental demand (*r* = −0.34; *p* = 0.04) and physical demand (*r* = −0.38; *p* = 0.02) correlated with peak amplitude at the Fz channel. The performance item of the NASA-TLX correlated with peak P3 latency at Fz (*r* = −0.39; *p* = 0.02). Finally, frustration levels correlated with peak P3 amplitude at channel Cz (*r* = −0.34; *p* = 0.04).

2-back: No correlations were found between NASA-TLX total scores and ERP measures for the 2-back. On the item level, physical demand (*r* = −0.37; *p* = 0.03) and temporal demand (*r* = −0.37; *p* = 0.03) correlated with P3 peak amplitude at channel Fz.

### 3.5. Convergent Validity of ICA

0-back: No significant correlations were found between ICA and ERP.

1-back: A larger right ICA mean correlated significantly with increased P3 peak latency at Fz (*r* = 0.32; *p* = 0.049) and at Cz (*r* = 0.33; *p* = 0.048) in the 1-back test. Likewise, a larger left ICA mean correlated with increased P3 peak latency at Pz (*r* = 0.35; *p* = 0.03) and with larger P3 peak amplitude at Cz (*r* = 0.32; *p* = 0.048).

2-back: A larger left ICA mean correlated with decreased P3 peak latency in Pz (*r* = −0.34; *p* = 0.04) in the 2-back test.

### 3.6. Correlation between ICA and NASA-TLX

0-back: No significant correlations were found between ICA and total NASA-TLX scores. On the item level, the right ICA mean correlated with the performance scores of the NASA-TLX (*r* = 0.34; *p* = 0.04).

1-back: No correlations were found between ICA and total or item scores of the NASA-TLX.

2-back: No correlations were found between ICA and total NASA-TLX scores. On the item level, strong correlations were found between the left ICA mean and physical demand in the NASA-TLX (*r* = 0.52; *p* = 0.001). Right ICA mean scores correlated with frustration levels in the NASA-TLX (*r* = 0.40; *p* = 0.02).

## 4. Discussion

Our results showed that pupillary response, transformed to an Index of Cognitive Activity (ICA), provides fair to good test–retest reliability as a measure of real-time cognitive workload in older adults with and without cognitive impairments. Subjective measures, such as the NASA-TLX, offer even better reliability in assessing cognitive workload in older adults. Moderate correlations were found between these two measures and the P3 ERP.

The ease of use of the NASA-TLX has resulted in its application in the diverse fields of aviation, military, human–machine interaction, driving, and medicine [7]. Despite the vast literature, few studies have reported on the test–retest reliability of the NASA-TLX in healthy adults and none in older adults with or without cognitive impairments. Battiste and Bortolussi reported strong test–retest reliability (*r* = 0.77) of the NASA-TLX in airborne pilots. Hart and Staveland found a correlation of 0.83 in NASA scores administered at baseline and at a four-week follow-up assessment in healthy adults [5]. Xiao reported a test–retest reliability of 0.75 in mental health workers [59]. These correlation coefficients are slightly higher than those found in our study (ranging between 0.55 and 0.68), which may be because our study focused on older adults with a wide range of cognitive ability. Previous studies have shown a potential confounding effect of cognitive impairment on the reliability of EEG ERP [9,60].

However, Pearson correlation coefficients tend to overestimate the true test–retest reliability. We extended the correlation analyses with intra-class correlation coefficients (ICCs), Bland–Altman plots, and minimal detectable change (MDC) calculations. ICC values provide a single measure of the magnitude of agreement while accounting for the differences in test moments along with the correlation between test moments. The ICCs showed good to excellent reliability for the NASA-TLX and no signs of systematic bias across the two test moments. The ICCs were higher and the score range was smaller (0.71–0.81) than those reported in a previous study (range 0.34–0.80) that taxed the mental and physical efforts of simulated manufacturing tasks in 24 college engineering students [50]. The ICC values were consistent across the groups of participants. None of the aforementioned studies provided a graphical representation of the measurement error across the two test moments. The Bland–Altman graphs showed relatively large limits of agreement, with no evidence of test or practice effect in all three tests. The MDC calculations showed changes of 15% to 25% of the total NASA-TLX scale scores to represent true change beyond measurement error. Taken together, these results indicate that subjective self-recall of cognitive workload is reliable across the spectrum of cognitive aging and has the potential to be used as a measure of attention allocation in this population.

Likewise, pupillometry has been used for over five decades as a measure of cognitive workload in the domains of psychophysiology, cognitive neuroscience, and human factor engineering, such as aviation or driving. Only recently has pupillometry received attention in the medical field as a potential marker of disease progression in adults with AD, Parkinson’s disease, and breast cancer [15,21,29,32]. This rekindled interest in pupillary response to cognitive workload as a marker of cognitive decline warrants an investigation of its psychometric properties. Overall, the ICA produced fair to good reliability scores, ranging between 0.56 and 0.78. These ICCs are in the same range as the reliability of our convergent measure, the P3 ERP component [9]. A comparison of reliability with other measures of pupillary response is complicated by the type of extraction (TEPR versus ICA), the type of task, and the population of interest. The closest object for comparison is a study by Kahya et al. that estimated the reliability of ICA during postural demanding tasks in Parkinson’s disease [61]. The ICCs in that study ranged between 0.74 and 0.93—higher than those reported in the current study. However, the retest study was done within hours after baseline assessment, and, therefore, this study did not take into account day-to-day fluctuations of cognitive functions within individuals. The ICC values of the ICA were consistent across groups. However, two unreliable ICC values were calculated for ICA for the 0-back and 2-back tests in the MCI/AD group. Cognitive impairments may potentially affect the reliability of neurophysiological measures [9]. The Bland–Altman plots revealed no systematic bias of ICA across test moments, except for the mean ICA of the left eye during the 0-back test. This negative value may be interpreted as a slight tendency towards a practice effect. The MDC values showed that a change of between 20% and 25% of the total scale score is needed to produce an effect that cannot be attributed to measurement error. These results suggest that ICA provides a stable measure of cognitive workload during cognitive testing in older adults with and without cognitive impairments.

NASA-TLX and ICA correlated only moderately with P3 ERP. A previous study demonstrated a strong correlation (*r* = −0.70) between peak ICA values and ERP P3 latency in healthy young adults [62]. A comparison of our results with this study is complicated since different ICA metrics (mean versus peak), ERP measures (amplitude versus peak), and populations (older versus younger) were used. In addition, in this study we used a measure of working memory whereas the other study used a cognitive-motor interference balance task. The *n*-back test is a multi-domain cognitive assessment rather than a single-domain test of working memory. These multidomain processes involved with the *n*-back test may explain the moderate correlations between the ICA and P3 ERP. An alternative explanation is that cognitive workload represents several dimensions of mental, physical, and temporal demand, along with effort, performance, and frustration. It may be that the ICA and P3 ERP measure overlapping and distinct constructs of cognitive workload. We found that mental demand, physical demand, temporal demand, performance, and frustration correlated significantly, yet weakly, with P3 ERP measures. Only physical demand, performance, and frustration correlated with ICA. In a previous study, ICA was found to correlate with the mental demand item of the NASA-TLX in people with Parkinson’s disease [61]. Future studies should continue to unravel the physiological substrates of cognitive workload. It also remains unclear why left and right ICA values produced different correlations. While some studies have suggested a lateralization effect of hemispheric function on pupillary response [63], in this case, the differences are likely due to measurement error.

To our knowledge, this is the first study evaluating the psychometric properties of subjective and objective measures of cognitive workload in a group of older adults with a heterogeneous profile of cognitive ability. The lack of detailed cognitive testing at the time of the EEG scan and the large time interval since their PET scan represents a limitation of this study. For example, some participants with preclinical AD may have developed cognitive symptoms since their last PET scan, and some participants with the clinical label of MCI may have converted to AD by the time of their EEG scan. The small sample size warrants caution in extrapolating the results to the older adult population. Finally, we did not establish the reliability of ERP in other cognitive domains known to deteriorate in older age, such as memory and language, and this remains an opportunity for further investigation. Future research should also investigate the added value of cognitive workload measures in the diagnosis, monitoring, and treatment of individuals at risk of dementia.

## 5. Conclusions

Our current results show that the NASA-TLX and ICA are reliable in older adults with and without cognitive impairment. The NASA-TLX in particular can be considered in the assessment of cognitive workload since the scale is short and easy to administer. The lack of strong correlation with the P3 ERP measure of cognitive workload may be due to the multidimensionality of the construct. Further research is needed to understand the physiological underpinnings of cognitive workload in older adults before these measures can be considered biomarkers of cognitive decline.

## Figures and Tables

**Table 1:** Comparison of NASA-TLX and ICA at baseline and at a two-week follow-up (*n* = 38).

Variable	Baseline	Follow-up	Pearson *r*	ICC, (95% CI)	MDC_90_
0-back, NASA-TLX	19.51 (15.95)	21.98 (17.89)	0.68 ^a^	0.81 (0.61–0.90) ^a^	15.82
0-back, mean ICA L	0.33 (0.14)	0.24 (0.15)	0.46 ^b^	0.63 (0.26–0.83) ^b^	0.20
0-back, mean ICA R	0.27 (0.17)	0.28 (0.16)	0.55 ^b^	0.70 (0.40–0.85) ^a^	0.22
1-back, NASA-TLX	28.24 (17.80)	27.22 (16.96)	0.60 ^a^	0.78 (0.57–0.89) ^a^	19.37
1-back, mean ICA L	0.29 (0.17)	0.27 (0.14)	0.58 ^a^	0.73 (0.47–0.86) ^a^	0.20
1-back, mean ICA R	0.25 (0.16)	0.30 (0.15)	0.39 ^c^	0.56 (0.12–0.78) ^b^	0.25
2-back, NASA-TLX	50.92 (19.41)	50.61 (19.13)	0.55 ^b^	0.71 (0.42–0.85) ^a^	24.33
2-back, mean ICA L	0.25 (0.14)	0.23 (0.16)	0.50 ^b^	0.64 (0.29–0.82) ^a^	0.24
2-back, mean ICA R	0.24 (0.18)	0.25 (0.15)	0.45 ^b^	0.62 (0.20–0.82) ^b^	0.25

Abbreviations: CI, confidence interval; ICA, Index of Cognitive Activity; ICC, intraclass correlation coefficient; L, left, NASA-TLX, National Aeronautics and Space Administration Task Load Index; MDC, minimal detectable difference; R, right. ^a^ *p* < 0.0001, ^b^ *p* < 0.01, ^c^ *p* < 0.05.

## Supplementary Materials

The following are available online at https://www.mdpi.com/2076-3425/10/12/994/s1, Table S1: IntraClass Correlation Coefficients (ICC) of NASA-TLX and ICA of the Three Subgroups.

## References

[1] Cowan N. (2010). The magical mystery four: How is working memory capacity limited, and why?. Curr. Dir. Psychol. Sci..

[2] Kahneman D. (1973). Attention and Effort.

[3] Bruya B., Tang Y.-Y. (2018). Is attention really effort? Revisiting Daniel Kahneman’s influential 1973 book Attention and effort. Front. Psychol..

[4] Ranchet M., Morgan J.C., Akinwuntan A.E., Devos H. (2017). Cognitive workload across the spectrum of cognitive impairments: A systematic review of physiological measures. Neurosci. Biobehav. Rev..

[5] Hart S.G., Staveland L.E., Hancock P.A., Meshkati N. (1988). Development of NASA-TLX (Task Load Index): Results of empirical and theoretical research. Advances in Psychology.

[6] Dias R.D., Ngo-Howard M.C., Boskovski M.T., Zenati M.A., Yule S.J. (2018). Systematic review of measurement tools to assess surgeons’ intraoperative cognitive workload. Br. J. Surg..

[7] Hart S.G. (2006). Nasa-task load index (NASA-TLX); 20 years later. Proc. Hum. Fact. Ergon. Soc. Annu. Meet..

[8] Tubbs-Cooley H.L., Mara C.A., Carle A.C., Gurses A.P. (2018). The NASA Task Load Index as a measure of overall workload among neonatal, paediatric and adult intensive care nurses. Intensiv. Crit. Care Nurs..

[9] Devos H., Burns J.M., Liao K., Ahmadnezhad P., Mahnken J.D., Brooks W.M., Gustafson K. (2020). Reliability of P3 event-related potential during working memory across the spectrum of cognitive aging. Front. Aging Neurosci..

[10] Bopp K.L., Verhaeghen P. (2018). Aging and n-back performance: A meta-analysis. J. Gerontol. Ser. B.

[11] Schmiedek F., Li S.-C., Lindenberger U. (2009). Interference and facilitation in spatial working memory: Age-associated differences in lure effects in the n-back paradigm. Psychol. Aging.

[12] Miller K., Price C., Okun M., Montijo H., Bowers D. (2009). Is the N-Back task a valid neuropsychological measure for assessing working memory?. Arch. Clin. Neuropsychol..

[13] Ahmadlou M., Adeli A., Bajo R., Adeli H. (2014). Complexity of functional connectivity networks in mild cognitive impairment subjects during a working memory task. Clin. Neurophysiol..

[14] Galluzzi S., Nicosia F., Geroldi C., Alicandri A., Bonetti M., Romanelli G., Zulli R., Frisoni G.B. (2009). Cardiac autonomic dysfunction is associated with white matter lesions in patients with mild cognitive impairment. J. Gerontol. Ser. A Biol. Sci. Med. Sci..

[15] Granholm E., Panizzon M.S., Elman J.A., Jak A.J., Hauger R.L., Bondi M.W., Lyons M.J., Franz C.E., Kremen W.S. (2017). Pupillary responses as a biomarker of early risk for Alzheimer’s Disease. J. Alzheimer’s Dis..

[16] Braak H., Thal D.R., Ghebremedhin E., Del Tredici K. (2011). Stages of the pathologic process in Alzheimer Disease: Age categories from 1 to 100 years. J. Neuropathol. Exp. Neurol..

[17] Wilson R.S., Nag S., Boyle P.A., Hizel L.P., Yu L., Buchman A.S., Schneider J.A., Bennett D.A. (2013). Neural reserve, neuronal density in the locus ceruleus, and cognitive decline. Neurology.

[18] Chandler D., Jensen P., McCall J.G., E Pickering A., Schwarz L.A., Totah N.K. (2019). Redefining noradrenergic neuromodulation of behavior: Impacts of a modular locus coeruleus architecture. J. Neurosci..

[19] Samuels E.R., Szabadi E. (2008). Functional neuroanatomy of the noradrenergic locus coeruleus: Its roles in the regulation of arousal and autonomic function part I: Principles of functional organisation. Curr. Neuropharmacol..

[20] Beatty J., Lucero-Wagoner B. (2000). The pupillary system. Handbook of Psychophysiology.

[21] Kremen W.S., Panizzon M.S., Elman J.A., Granholm E.L., Andreassen O.A., Dale A.M., Gillespie N.A., Gustavson D.E., Logue M.W., Lyons M.J. (2019). Pupillary dilation responses as a midlife indicator of risk for Alzheimer’s disease: Association with Alzheimer’s disease polygenic risk. Neurobiol. Aging.

[22] Alnaes D., Sneve M.H., Espeseth T., Endestad T., Van De Pavert S.H.P., Laeng B. (2014). Pupil size signals mental effort deployed during multiple object tracking and predicts brain activity in the dorsal attention network and the locus coeruleus. J. Vis..

[23] Mathur A., Gehrmann J., Atchison D.A. (2013). Pupil shape as viewed along the horizontal visual field. J. Vis..

[24] Marshall S. The Index of Cognitive Activity: Measuring cognitive workload. Proceedings of the IEEE 7th Conference on Human Factors and Power Plants, Institute of Electrical and Electronics Engineers (IEEE).

[25] Duchowski A.T., Krejtz K., Kreitz I., Biele C., Niedzielska A., Kiefer P., Raubal M., Giannopoulos I. The index of pupillary activity. Proceedings of the 2018 CHI Conference on Human Factors in Computing Systems.

[26] Stark L., Campbell F.W., Atwood J. (1958). Pupil unrest: An example of noise in a biological servomechanism. Nat. Cell Biol..

[27] Demberg V., Sayeed A. (2016). The frequency of rapid pupil dilations as a measure of linguistic processing difficulty. PLoS ONE.

[28] Devos H., Akinwuntan A.E., Alissa N., Morohunfola B., Lynch S. (2020). Cognitive performance and cognitive workload in multiple sclerosis: Two different constructs of cognitive functioning?. Mult. Scler. Relat. Disord..

[29] Kahya M., Moon S., Lyons K.E., Pahwa R., Akinwuntan A.E., Devos H. (2018). Pupillary response to cognitive demand in Parkinson’s Disease: A pilot study. Front. Aging Neurosci..

[30] Moon S., Kahya M., Lyons K.E., Pahwa R., Akinwuntan A., Devos H. (2020). Cognitive workload during verbal abstract reasoning in Parkinson’s disease: A pilot study. Int. J. Neurosci..

[31] Myers J.S., Alissa N., Mitchell M., Dai J., He J., Moon S., O’Dea A., Klemp J., Kurylo M., Akinwuntan A. (2020). Pilot feasibility study examining pupillary response during driving simulation as a measure of cognitive load in breast cancer survivors. Oncol. Nurs. Forum.

[32] Myers J.S., Kahya M., Mitchell M., Dai J., He J., Moon S., Hamilton K., Valla M., O’Dea A., Klemp J. (2019). Pupillary response: Cognitive effort for breast cancer survivors. Support. Care Cancer.

[33] Ranchet M., Orlosky J., Morgan J., Qadir S., Akinwuntan A.E., Devos H. (2017). Pupillary response to cognitive workload during saccadic tasks in Parkinson’s disease. Behav. Brain Res..

[34] Wang C., Gao J., Li M., Qi H., Zhao T., Zhang B., Zhou C., Fang S. (2019). Association of cognitive impairment and mood disorder with event-related potential P300 in patients with cerebral small vessel diseases. Neuro Endocrinol. Lett..

[35] Jervis B.W., Bigan C., Besleaga M., Jervis M.W. (2019). New-Onset Alzheimer’s Disease and normal subjects 100% differentiated by pam. J. Alzheimer’s Dis. Other Dement..

[36] Ghani U., Signal N., Niazi I.K., Taylor D. (2020). ERP based measures of cognitive workload: A review. Neurosci. Biobehav. Rev..

[37] Hedges D., Janis R., Mickelson S., Keith C., Bennett D., Brown B.L. (2016). P300 amplitude in Alzheimer’s Disease. Clin. EEG Neurosci..

[38] Pedroso R.V., Fraga F.J., Icassatti Corazza D., Almeida Andreatto C.A., Gomes de Melo Coelho F., Riani Costa J.L., Ferreira Santos-Galduróz R. (2012). Latência e amplitude do P300 auditivo na doença de Alzheimer: Uma revisão sistemática. Braz. J. Otorhinolaryngol..

[39] Lee M.-S., Lee S.-H., Moon E.-O., Moon Y.-J., Kim S., Kim S.-H., Jung I.-K. (2013). Neuropsychological correlates of the P300 in patients with Alzheimer’s disease. Prog. Neuro-Psychopharmacol. Biol. Psychiatry.

[40] Chapman R.M., McCrary J.W., Gardner M.N., Sandoval T.C., Guillily M.D., Reilly L.A., DeGrush E. (2011). Brain ERP components predict which individuals progress to Alzheimer’s Disease and which do not. Neurobiol. Aging.

[41] Bennys K., Portet F., Touchon J., Rondouin G. (2007). Diagnostic value of event-related evoked potentials N200 and P300 subcomponents in early diagnosis of Alzheimer’s Disease and mild cognitive impairment. J. Clin. Neurophysiol..

[42] Rossini P., Di Iorio R., Vecchio F., Anfossi M., Babiloni C., Bozzali M., Bruni A., Cappa S., Escudero J., Fraga F. (2020). Early diagnosis of Alzheimer’s disease: The role of biomarkers including advanced EEG signal analysis. Report from the IFCN-sponsored panel of experts. Clin. Neurophysiol..

[43] Boutros N., Torello M.W., Burns E.M., Wu S.-S., Nasrallah H.A. (1995). Evoked potentials in subjects at risk for Alzheimer’s Disease. Psychiatry Res..

[44] Nieuwenhuis S., De Geus E.J., Aston-Jones G. (2011). The anatomical and functional relationship between the P3 and autonomic components of the orienting response. Psychophysiology.

[45] Murphy P.R., Robertson I.H., Balsters J.H., O’Connell R.G. (2011). Pupillometry and P3 index the locus coeruleus-noradrenergic arousal function in humans. Psychophysiology.

[46] Sperling A.R., Aisen P.S., Beckett L.A., Bennett D.A., Craft S., Fagan A.M., Iwatsubo T., Jack C.R., Kaye J., Montine T.J. (2011). Toward defining the preclinical stages of Alzheimer’s disease: Recommendations from the National Institute on Aging-Alzheimer’s Association workgroups on diagnostic guidelines for Alzheimer’s disease. Alzheimer’s Dement..

[47] Vidoni E.D., Yeh H.-W., Morris J.K., Newell K.L., Alqahtani A., Burns N.C., Burns J.M., Billinger S.A. (2016). Cerebral β-Amyloid angiopathy is associated with earlier dementia onset in Alzheimer’s Disease. Neurodegener. Dis..

[48] Nasreddine Z.S., Phillips N.A., Bedirian V., Charbonneau S., Whitehead V., Collin I., Cummings J.L., Chertkow H. (2005). The Montreal cognitive assessment, MoCA: A brief screening tool for mild cognitive impairment. J. Am. Geriatr. Soc..

[49] Gevins A.S., Smith M.E., McEvoy L.K., Ilan A.B., Chan C.S., Jiang A., Sam-Vargas L., Abraham G. (2011). A cognitive and neurophysiological test of change from an individual’s baseline. Clin. Neurophysiol..

[50] Ikuma L.H., Nussbaum M.A., Babski-Reeves K.L. (2009). Reliability of physiological and subjective responses to physical and psychosocial exposures during a simulated manufacturing task. Int. J. Ind. Ergon..

[51] Marshall S.P. (2000). Method and Apparatus for Eye Tracking and Monitoring Pupil Dilation to Evaluate Cognitive Activity. U.S. Patent.

[52] Vogels J., Demberg V., Kray J. (2018). The index of cognitive activity as a measure of cognitive processing load in dual task settings. Front. Psychol..

[53] Delorme A., Makeig S. (2004). EEGLAB: An open source toolbox for analysis of single-trial EEG dynamics including independent component analysis. J. Neurosci. Methods.

[54] Lopez-Calderon J., Luck S.J. (2014). ERPLAB: An open-source toolbox for the analysis of event-related potentials. Front. Hum. Neurosci..

[55] Shrout P.E., Fleiss J.L. (1979). Intraclass correlations: Uses in assessing rater reliability. Psychol. Bull..

[56] Cicchetti D.V. (1994). Guidelines, criteria, and rules of thumb for evaluating normed and standardized assessment instruments in psychology. Psychol. Assess..

[57] Bland J.M., Altman D.G. (1986). Statistical methods for assessing agreement between two methods of clinical measurement. Lancet.

[58] Donoghue D., Stokes E.K. (2009). Physiotherapy research and older people (PROP) group how much change is true change? The minimum detectable change of the Berg Balance Scale in elderly people. J. Rehabil. Med..

[59] Xiao Y.M., Wang Z.-M., Wang M.-Z., Lan Y.-J. (2005). The appraisal of reliability and validity of subjective workload assessment technique and NASA-task load index. Zhonghua Lao Dong Wei Sheng Zhi Ye Bing Za Zhi,.

[60] Lew H.L., Gray M., Poole J.H. (2007). Temporal stability of auditory event-related potentials in healthy individuals and patients with traumatic brain injury. J. Clin. Neurophysiol..

[61] Kahya M., Lyons K.E., Pahwa R., Akinwuntan A.E., He J., Devos H. (2020). Reliability and validity of pupillary response during dual-task balance in Parkinson Disease. Arch. Phys. Med. Rehabil..

[62] Kahya M., Liao K., Gustafson K., Akinwuntan A., Devos H. (2019). Validation of pupillary response against EEG during dual-tasking postural control. Arch. Phys. Med. Rehabil..

[63] Kim M., Barrett A.M., Heilman K.M. (1998). Lateral asymmetries of pupillary responses. Cortex.

