# Supplementary material for: Psychometric Properties of NASA-TLX and Index of Cognitive Activity as Measures of Cognitive Workload in Older Adults
Source: Brain Sci. 2020 Dec 16;10(12):994. doi: 10.3390/brainsci10120994 (PMC7766152; doi:10.3390/brainsci10120994)
Supplement: Supplementary file 1 [file brainsci-10-00994-s001.pdf]

Supplementary File

Table S1. IntraClass Correlation Coefficients (ICC) of NASA-TLX and ICA of the Three Subgroups.

	**Aβ− (*n* = 16)**	**Aβ+ (*n* = 16)**	**MCI/AD (*n* = 6)**
Variable	ICC, (95% CI)	ICC, (95% CI)	ICC, (95% CI)
0-back, NASA-TLX	0.70 (0.06 − 0.90) [c]	0.95 (0.83 − 0.98) [a]	0.87 (−0.18 − 0.99) [b]
0-back, mean ICA L	0.79 (0.33 − 0.93) [b]	0.44 (−0.68 − 0.81)	0.06 (−7.94 − 0.90)
0-back, mean ICA R	0.80 (0.34 − 0.94) [b]	0.55 (−0.35 − 0.85)	0.45 (−4.24 − 0.94)
1-back, NASA-TLX	0.50 (−0.057 − 0.84)	0.83 (0.47 − 0.95) [b]	0.88 (0.13 − 0.98) [b]
1-back, mean ICA L	0.64 (−0.08 − 0.88) [c]	0.88 (0.62 − 0.95) [a]	0.74 (−1.43 − 0.97)
1-back, mean ICA R	0.63 (−0.15 − 0.88) [c]	0.42 (−0.65 − 0.80)	0.91 (0.14 − 0.99) [b]
2-back, NASA-TLX	0.59 (−0.39 − 0.87)	0.73 (0.20 − 0.91) [b]	0.87 (0.03 − 0.98) [c]
2-back, mean ICA L	0.63 (−0.10 − 0.88) [b]	0.77 (0.32 − 0.92) [b]	−2.44 (−32.04 − 0.64)
2-back, mean ICA R	0.60 (−0.41 − 0.88)	0.66 (−0.07 − 0.89) [a]	0.67 (−2.19 − 0.97)

Aβ−, cognitively normal, non-elevated; Aβ+, cognitively normal, elevated; CI, confidence interval; ICA, Index of Cognitive Activity; MCI/AD, mild cognitive impairment or Alzheimer's disease; NASA-TLX, NASA-Task Load Index. [a] $p < 0.0001$, [b] $p < 0.01$, [c] $p < 0.05$.
